# Object Detection Method for Grasping Robot Based on Improved YOLOv5

**DOI:** 10.3390/mi12111273

**Published:** 2021-10-20

**Authors:** Qisong Song, Shaobo Li, Qiang Bai, Jing Yang, Xingxing Zhang, Zhiang Li, Zhongjing Duan

**Affiliations:** 1 College of Mechanical Engineering, Guizhou University, Guiyang 550025, China; gs.qssong18@gzu.edu.cn (Q.S.); cme.qbai18@gzu.edu.cn (Q.B.); jyang23@gzu.edu.cn (J.Y.); 11136827324@163.com (Z.L.); 2 State Key Laboratory of Public Big Data, Guizhou University, Guiyang 550025, China; xingxingzhang_star@163.com; 3 Key Laboratory of Advanced Manufacturing Technology of Ministry of Education, Guizhou University, Guiyang 550025, China; duan_zhongjing@163.com

**Keywords:** grasping robot, object detection, improved YOLOv5 network, hand-eye calibration method, convolutional neural network

## Abstract

In the industrial field, the anthropomorphism of grasping robots is the trend of future development, however, the basic vision technology adopted by the grasping robot at this stage has problems such as inaccurate positioning and low recognition efficiency. Based on this practical problem, in order to achieve more accurate positioning and recognition of objects, an object detection method for grasping robot based on improved YOLOv5 was proposed in this paper. Firstly, the robot object detection platform was designed, and the wooden block image data set is being proposed. Secondly, the Eye-In-Hand calibration method was used to obtain the relative three-dimensional pose of the object. Then the network pruning method was used to optimize the YOLOv5 model from the two dimensions of network depth and network width. Finally, the hyper parameter optimization was carried out. The simulation results show that the improved YOLOv5 network proposed in this paper has better object detection performance. The specific performance is that the recognition precision, recall, mAP value and F1 score are 99.35%, 99.38%, 99.43% and 99.41% respectively. Compared with the original YOLOv5s, YOLOv5m and YOLOv5l models, the mAP of the YOLOv5_ours model has increased by 1.12%, 1.2% and 1.27%, respectively, and the scale of the model has been reduced by 10.71%, 70.93% and 86.84%, respectively. The object detection experiment has verified the feasibility of the method proposed in this paper.

## 1. Introduction

With the rapid development of computer vision technology, computer vision tasks such as object detection and object segmentation are being widely applied in many fields of life [1,2,3,4,5,6]. For robot grasping tasks, object detection aims to locate and recognize objects, which helps robots to be able to pick up objects more accurately [7]. Therefore, the accuracy of object detection results is very important in the field of robot grasping.

The current object detection algorithm is mainly based on a deep learning object detection algorithm, which includes a one-stage object detection algorithm and a two-stage object detection algorithm [8,9]. The detection speed of the one-stage object detection algorithm is faster than the two-stage object detection algorithm. The one-stage object detection algorithm mainly includes SSD [10], YOLO [11], YOLOv2 [12], YOLOv3 [13], YOLOv4 [14] and YOLOv5 [15]. The two-stage object detection algorithm mainly includes R-CNN [16], Fast R-CNN [17] and Faster R-CNN [18]. In recent times, robots are widely applied in industrial handling and grasping, but there are some problems such as the single working mode, impacts of sensitive environments, inaccurate positioning, and low recognition efficiency. The object detection algorithm based on deep learning has powerful autonomous learning ability, which can make the robot locate and recognize objects more accurately in complex environments.

Object detection algorithms were widely used in agriculture [19], medical treatment [20], industry [21], transportation [22] and other fields. In [23], a multi-target matching tracking method based on YOLO is proposed, which can be used to detect the passengers flow on and off buses. In [24], a target detection method based on an improved YOLO network was proposed, which detected spilled loads on freeways. In [25], a license plate recognition method based on deep learning was proposed; it detected license plates and accurately recognized characters in complex and diverse environment. In [26], a target detection method based on deep learning was proposed; it improved the accuracy of safety helmet wearing detection. Based on Fast R-CNN, the researchers carried out face contour detection [27] and automatic detection of ripe tomatoes [28]. Meanwhile, Faster R-CNN was also applied to medical anatomy [29] and crop identification [30]. However, industrial robots such as electric welding robots, picking robots [31], grasping robots [32], handling robots [33] and multi-robots [34] all use point-to-point basic vision technology.

It can be seen from the above research that the object detection algorithm based on deep learning was studied more and achieved good results in the fields of transportation, medical treatment, agriculture and so on, but there is less research in the field of robot application, and more extensive and in-depth research is needed. Therefore, we propose an improved YOLOv5 method to improve the flexibility of robot recognition and the accuracy of localization.

The main contributions of our work can be summarized as follows:

(1) This study designs and builds a robot object detection platform, and adopts the hand-eye calibration method to improve the accuracy of robot object positioning.
(2) This study proposes an improved YOLOv5 object detection algorithm. Compared with the YOLOv5 series, the proposed algorithm can effectively improve the precision and recall of robot object recognition.
(3) This study adopts the network pruning method to design a lighter and more efficient YOLOv5 network model, which is suitable for object recognition of grasping robots, and has great potential to be deployed in industrial equipment.
(4) In order to prove the feasibility of our method, we have carried out experimental verification of object detection by using grasping robot.

The main structure of this paper is as follows: Section 2 mainly describes the experimental platform and experimental process for grasping robot object detection. Section 3 designs and makes the wooden block image datasets and introduces the hand-eye calibration method. Section 4 proposes an improved YOLOv5 network model which is applied for grasping robot object detection. Section 5 presents the simulation and experimental results. Section 6 concludes the paper and recommendations for future works.

## 2. Experimental Device 

### 2.1. Experimental Platform

**Figure 1:**
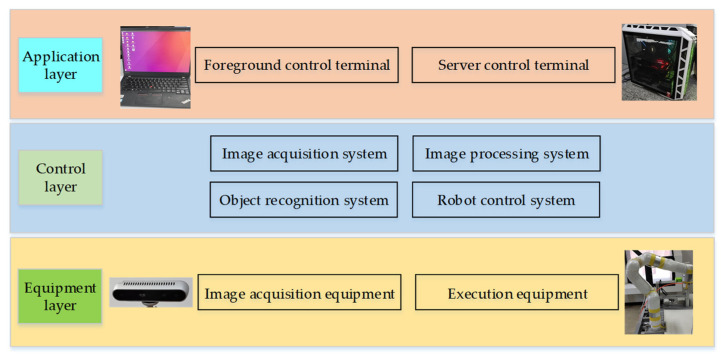
Robot object detection system.

The robot object detection system based on machine vision is composed of an application layer, a control layer and an equipment layer, which is shown in Figure 1. The application layer is composed of the foreground control terminal and the server control terminal. The control layer is composed of an image acquisition system, an image processing system, an object recognition system, and a robot control system. The equipment layer is composed of image acquisition equipment and execution equipment.

**Figure 2:**
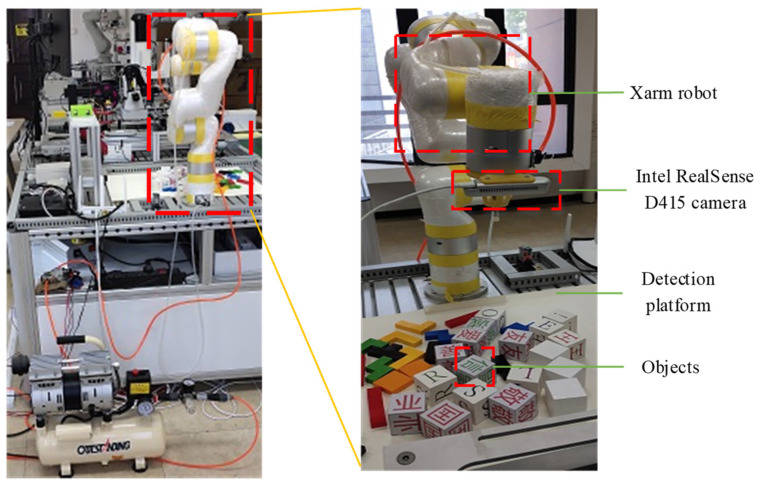
Robot object detection platform.

This paper studied the robot object detection method based on machine vision, the robot object detection platform is designed and built, which is shown in Figure 2. It mainly includes an Xarm robot, a detection platform, an Intel RealSense D415 camera and a server. The Xarm robot is a cost-effective lightweight programmable robot, which is composed of a robot manipulator, a control cabinet, a signal cable, a power supply cable and other components. The Intel RealSense D415 camera is equipped with a D410 depth sensor, which has better resolution and higher accuracy. It can realize the conversion between the optical signal and the electrical signal, and then transmit the corresponding analog signal and digital signal to the server.

### 2.2. Experimental Process

**Figure 3:**
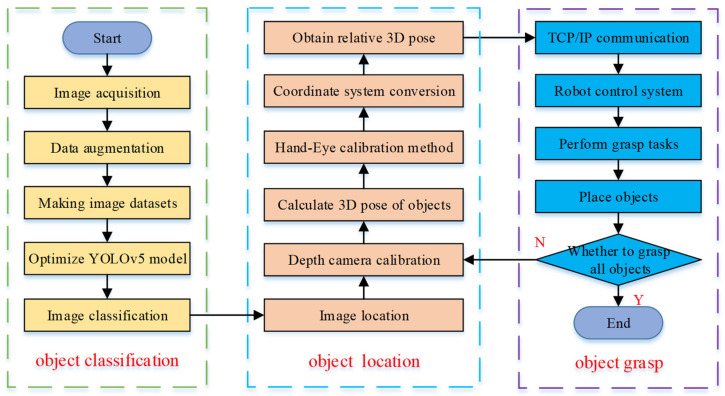
Flow chart of grasping robot object detection experiment.

Object detection tasks include object classification and object location, that is, judging the category of objects and obtaining the specific location of objects in space. Therefore, the grasping robot object detection experiment mainly includes three parts, that is, object classification, object location and object grasp. The overall experimental process is shown in Figure 3.

Object classification. Firstly, the object images were collected, and the collected object images were augmented to increase the image data. Secondly, the collected images and augmented images were made into datasets. Then, the YOLOv5 model was trained on the dataset. Finally, the object images were classified, and the accuracy and precision of object classification were improved.

Object location. Firstly, the object images were located, and the depth camera was used to obtain the depth information of the objects. Secondly, the three-dimensional pose of the object was calculated. Then, the Hand-Eye calibration method was used to obtain the relative pose conversion matrix between each coordinate system. Finally, the three-dimensional pose of the object relative to the robot was obtained through the pose conversion matrix.

Object grasp. Firstly, TCP/IP protocol was used to realize the communication between the host computer and robot [35]. Secondly, the three-dimensional pose was transmitted to the robot control system. Then, the robot control system controlled the robot to move and perform grasping tasks. Finally, the grasped object was placed in the set position in order.

## 3. Object Detection

### 3.1. Dataset

**Figure 4:**
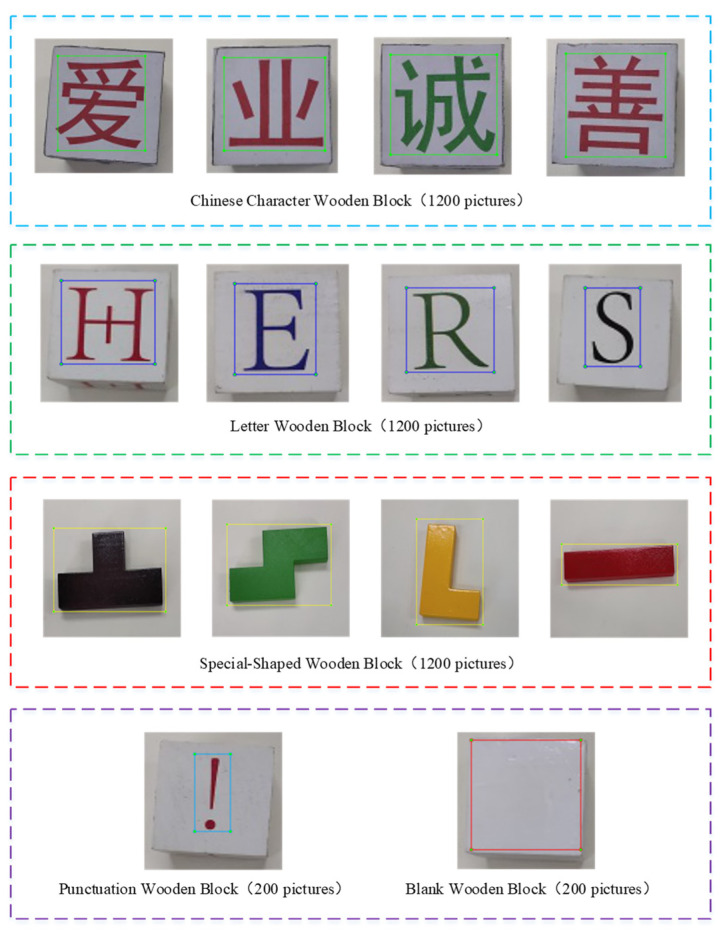
Wooden block image datasets.

In the study, the wooden blocks were used as research objects to simulate the industrial grasping task, and the wooden blocks were taken as the epitome of various industrial products. Wooden block image acquisition is the basis and the important link in the research of object detection methods for a grasping robot. In this experiment, the wooden block images were collected by the image acquisition system, and the collected wooden block image datasets are mainly divided into 5 categories, which is shown in Figure 4. Chinese character wooden blocks refer to industrial products with Chinese logos, letter wooden blocks refer to industrial products with English logos, special-shaped wooden blocks refer to industrial products with irregular shapes, punctuation wooden blocks refer to industrial products with defection, and blank wooden blocks refer to empty industrial products.

The object detection model based on deep learning was generated on the basis of a large amount of image data training. Therefore, we use data augmentation methods to expand the datasets [36]. Data augmentation manipulates datasets by rotating, flipping and clipping, so as to increase the number of datasets and prevent over-fitting. Where there are 1000 images collected by the image acquisition system, and additional 3000 sample images can be obtained after image augmentation operations. The quantity details of wooden block image datasets are shown in Table 1.

### 3.2. Hand-Eye Calibration Method

#### 3.2.1. Coordinate System Conversion

**Figure 5:**
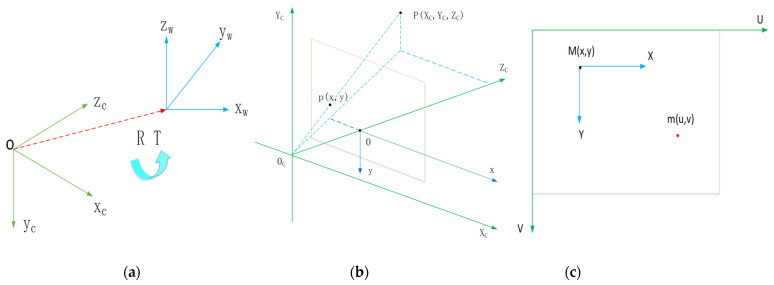
Coordinate system conversion relationship. (**a**) Conversion between world coordinate system and camera coordinate system; (**b**) Conversion between camera coordinate system and image coordinate system; (**c**) Conversion between image coordinate system and pixel coordinate system.

After the camera recognizes the object, it needs to be positioned first, analyze its three-dimensional posture, and then perform the grasping operation. When collecting image information, in order to ensure that the image features are the same as those of the objects, it is necessary to establish a stable relationship between the camera, the Xarm robot and the objects. Therefore, we adopted the hand-eye calibration method to achieve the positioning of the target object, which involves the conversion between various coordinate systems, and can provide positioning services for grasping tasks. The conversion relationship between coordinate systems is shown in Figure 5.

The conversion relationship between the world coordinate system and the camera coordinate system can be described as Equation (1):

(1) $$\left[\begin{array}{c} X_{C}\\ Y_{C}\\ Z_{C} \end{array}\right]=R\left[\begin{array}{c} X_{W}\\ Y_{W}\\ Z_{W} \end{array}\right]+T\Rightarrow \left[\begin{array}{c} X_{C}\\ Y_{C}\\ Z_{C}\\ 1 \end{array}\right]=\left[\begin{array}{cc} R & T\\ 0 & 1 \end{array}\right]\left[\begin{array}{c} X_{W}\\ Y_{W}\\ Z_{W}\\ 1 \end{array}\right]$$

where *R* represents the rotation matrix, and its structure is 3 × 3. *T* represents the translation matrix, and its structure is 3 × 1.

The conversion relationship between the camera coordinate system and the image coordinate system can be described as Equation (2):

(2) $$Z_{C}\left[\begin{array}{c} x\\ y\\ 1 \end{array}\right]=\left[\begin{array}{cccc} f & 0 & 0 & 0\\ 0 & f & 0 & 0\\ 0 & 0 & 1 & 0 \end{array}\right]\left[\begin{array}{c} X_{C}\\ Y_{C}\\ Z_{C}\\ 1 \end{array}\right]$$

where *f* is the camera focal length value obtained by camera calibration.

The conversion relationship between the image coordinate system and the pixel coordinate system can be described as Equation (3):

(3) $$\{\begin{array}{c} u=\frac{x}{dx}+u_{0}\\ v=\frac{y}{dy}+v_{0} \end{array}\Rightarrow \left[\begin{array}{c} u\\ v\\ 1 \end{array}\right]=\left[\begin{array}{ccc} \frac{1}{dx} & 0 & u_{0}\\ 0 & \frac{1}{dy} & v_{0}\\ 0 & 0 & 1 \end{array}\right]\left[\begin{array}{c} x\\ y\\ 1 \end{array}\right]$$

where, ($u_{0}$,$v_{0}$) is the coordinate origin, and dx and dy represent the pixel values of a point in the image on the *x*-axis and *y*-axis, respectively.

According to the above equations, the conversion relationship between the world coordinate system and the pixel coordinate system can be derived as follows:

(4) $$\left[\begin{array}{c} x\\ y\\ 1 \end{array}\right]=\left[\begin{array}{ccc} \frac{1}{dx} & 0 & u_{0}\\ 0 & \frac{1}{dy} & v_{0}\\ 0 & 0 & 1 \end{array}\right]\left[\begin{array}{cccc} f & 0 & 0 & 0\\ 0 & f & 0 & 0\\ 0 & 0 & 1 & 0 \end{array}\right]\left[\begin{array}{cc} R & T\\ 0 & 1 \end{array}\right]\left[\begin{array}{c} X_{W}\\ Y_{W}\\ Z_{W}\\ 1 \end{array}\right]=\left[\begin{array}{cccc} f_{x} & 0 & u_{0} & 0\\ 0 & f_{y} & v_{0} & 0\\ 0 & 0 & 1 & 0 \end{array}\right]\left[\begin{array}{cc} R & T\\ 0 & 1 \end{array}\right]\left[\begin{array}{c} X_{W}\\ Y_{W}\\ Z_{W}\\ 1 \end{array}\right]$$

(5) $$\left[\begin{array}{c} X_{W}\\ Y_{W}\\ 1 \end{array}\right]=R^{-1}\left({\left[\begin{array}{ccc} \frac{1}{dx} & 0 & u_{0}\\ 0 & \frac{1}{dy} & v_{0}\\ 0 & 0 & 1 \end{array}\right]}^{-1}\left[\begin{array}{c} x\\ y\\ 1 \end{array}\right]-T\right)/Z_{W}$$

#### 3.2.2. Eye-In-Hand

**Figure 6:**
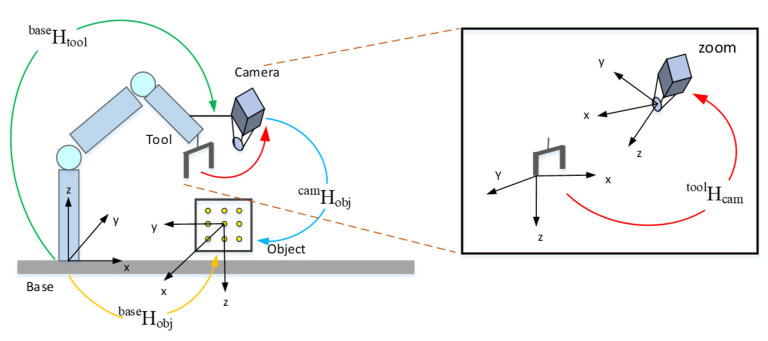
Eye-In-Hand.

Hand-Eye calibration methods are mainly divided into “Eye-In-Hand” [37] and “Eye-To-Hand”, where the manipulator and camera are equivalent to “hand” and “eye”, respectively. In this paper, we adopted the “Eye-In-Hand” calibration method, which is shown in Figure 6. It can be seen from the figure that the position matrix relationship of each coordinate system can be described as the Equation (6).

(6) Hobjbase=Htoolbase·Hcamtool·Hobjcam

where Hobjbase is the relative position matrix of the robot base and the objects, Htoolbase is the relative position matrix of the robot base and the robot gripping end, Hcamtool is the relative position matrix of the robot gripping end and the camera, and Hobjcam is the relative position matrix of the camera and the objects. 

## 4. Improved YOLOv5 Network

### 4.1. YOLOv5 Network

#### 4.1.1. Characteristics of YOLOv5 Network Structure

The YOLOv5 network is the latest product of YOLO, which has the advantages of high detection accuracy, fast detection speed and lightweight characteristics. There are mainly 4 models in YOLOv5, where YOLOv5x is the extended model, YOLOv5l is the benchmark model, and YOLOv5s and YOLOv5m are the preset simplified models. Their main differences are that the number of feature extraction modules and convolution kernels at specific locations of the network are different; the model size and the number of model parameters decrease in turn. 

The YOLOv5 network structure consists of the Input, Backbone network, and Neck network and Head, which is shown in Table 2. The Input terminal adopts Mosaic data augmentation, adaptive anchor, adaptive image scaling and so on. The Backbone network is a convolutional neural network [38] which aggregates different fine-grained images and forms image features. It is mainly composed of the focus module, CONV-BN-Leaky ReLU (CBL) module, CSP1_X module and other modules. The Neck network is a series of feature aggregation layers of mixed and combined image features, which is mainly used to generate FPN and PAN. It is mainly composed of the CBL module, Upsample module, CSP2_X module and other modules. The Head terminal takes GIoU_ Loss as the loss function of the bounding box.

#### 4.1.2. Bounding-Box Regression and Loss Function

**Figure 7:**
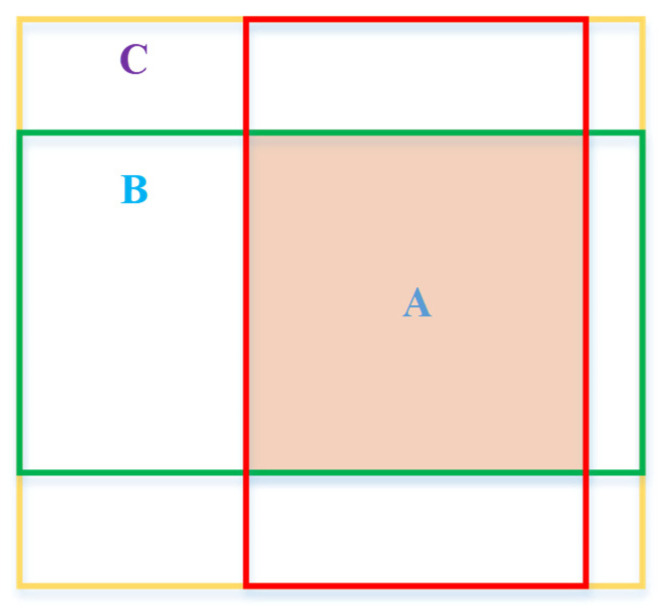
GIoU evaluation diagram.

In object detection, Intersection over Union (IoU) [39] is a standard for detecting object accuracy, which is used to measure the similarity between the predicted bounding box and the real bounding box, and can be described as Equation (7):

(7) $$\mathrm{IoU}=\frac{\mathrm{area}(\mathrm{box}(\mathrm{Pred})\;\cap \mathrm{box}(\mathrm{Truth}))}{\mathrm{area}(\mathrm{box}(\mathrm{Pred})\;\cup \mathrm{box}(\mathrm{Truth}))}$$

where the value range of IoU is [0, 1], which is a normalized index. However, when the two bounding boxes do not overlap or there are different ways of overlap, that is, the overlapping parts are the same, but the overlapping direction is different, the IoU is no longer reliable. Therefore, this paper takes Generalized IoU (GIoU) [40] as the evaluation index of the predicted bounding box, which can be represented by Figure 7. GIoU not only has the basic performance of IoU but also weakens the shortcomings of IoU, which can be described as the Equation (8):

(8) $$\mathrm{GIoU}=\frac{\left|A\cap B\right|}{\left|A\cup B\right|}-\frac{\left|C\backslash (A\cup B)\right|}{\left|C\right|}=IoU-\frac{\left|C\backslash (A\cup B)\right|}{\left|C\right|}$$

where *A* and *B* are two bounding boxes of arbitrary shapes, *C* is the smallest rectangular box that can completely contain *A* and *B*, and the value range of GIoU is [−1, 1].

In the object detection task, the loss function is usually used to describe the degree of difference between the predicted value and the real value of the model. The loss function of the YOLOv5 model includes three parts: bounding box regression loss, confidence loss and classification loss.

The loss function of bounding box regression is expressed as

(9) $$L_{\mathrm{GIoU}}=\sum_{i=0}^{S^{2}}\sum_{j=0}^{B}I_{i,j}^{\mathrm{obj}}\left[1-IoU+\frac{A^{c}-U}{A^{c}}\right]$$

where $s^{2}$ denotes the number of grids, and B denotes the number of bounding boxes in each grid; when an object exists in a bounding box, $I_{i,j}^{\mathrm{obj}}$ is equal to 1, otherwise it is 0.

The loss function of confidence is expressed as

(10) $$\begin{array}{ll} L_{\mathrm{conf}}= & -\sum_{i=0}^{S^{2}}\sum_{j=0}^{B}I_{i,j}^{\mathrm{obj}}\left[{\overset{⌢}{C}}_{i}^{j}\log(C_{i}^{j})+(1-{\overset{⌢}{C}}_{i}^{j})\log(1-C_{i}^{j})\right]\\ & -\lambda_{\mathrm{noobj}}\sum_{i=0}^{S^{2}}\sum_{j=0}^{B}I_{i,j}^{\mathrm{noobj}}\left[{\overset{⌢}{C}}_{i}^{j}\log(C_{i}^{j})+(1-C_{i}^{j})\log(1-{\overset{⌢}{C}}_{i}^{j})\right] \end{array}$$

where ${\overset{⌢}{C}}_{i}^{j}$ represents the prediction confidence of the *j*-th bounding box in the *i*-th grid, and $C_{i}^{j}$ represents the true confidence of the *j*-th bounding box in the *i*-th grid, and $\lambda_{\mathrm{noobj}}$ represents the confidence weight when no object exists in the bounding box.

The loss function of classification is expressed as

(11) $$L_{\mathrm{class}}=-\sum_{i=0}^{S^{2}}I_{i,j}^{\mathrm{noobj}}\sum_{c\in \mathrm{classes}}\left[{\overset{⌢}{P}}_{i}^{j}(c)\log(P_{i}^{j}(c))+(1-{\overset{⌢}{P}}_{i}^{j}(c))\log(1-P_{i}^{j}(c))\right]$$

where ${\overset{⌢}{P}}_{i}^{j}\left(c\right)$ represents the probability of predicting the detection object as category *c*, and $P_{i}^{j}\left(c\right)$ represents the probability of actually being category *c*.

According to the above equations, the total loss function is calculated and can be expressed as

(12) $$\mathrm{LOSS}=L_{\mathrm{GIoU}}+L_{\mathrm{conf}}+L_{\mathrm{class}}$$

In addition, this paper mainly evaluates the precision and recall of object detection. According to the confusion matrix [41], the precision, recall and mean average precision (mAP) can be described as follows:

(13) $$P=\frac{TP}{TP+FP}$$

(14) $$R=\frac{TP}{TP+FN}$$

(15) $$mAP=\frac{1}{C}\sum_{k=i}^{N}P\left(k\right)\Delta R\left(k\right)$$

where C represents the number of object categories, N represents the number of IoU thresholds, k is the IoU threshold, P(k) is the precision and R(k) is the recall.

### 4.2. Improvement of YOLOv5 Network

#### 4.2.1. Improvement of YOLOv5 Network Structure

**Figure 8:**
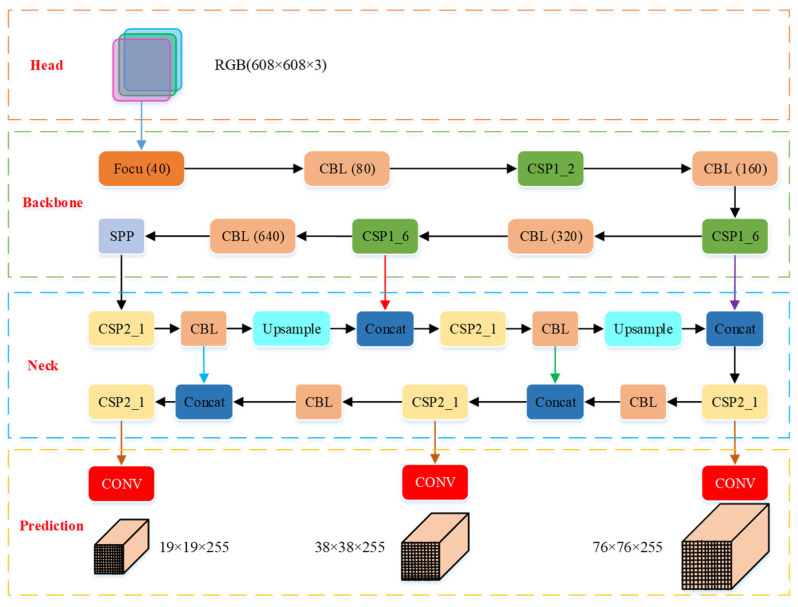
Architecture of improved YOLOv5 network.

The huge amount of calculation is a major obstacle to the industrialization of deep learning technology. For the research on the object detection method of grasping robots, reducing the amount of calculation and network storage space is the top priority of its optimization. Therefore, our method for optimizing the YOLOv5 model is model compression strategy, that is, using the network pruning method to obtain a lighter and more efficient YOLOv5 model. The network pruning method is mainly used to enhance the generalization performance of the network and to avoid over-fitting by reducing network parameters and structural complexity. In this paper, the improved YOLOv5 network architecture we propose is shown in Figure 8.

It can be seen from Figure 8 that the improved YOLOv5 network is mainly composed of four parts, where the Input terminal receives the collected datasets, the Backbone and Neck networks are the main part of network pruning, and the Prediction terminal provides the prediction results of the model.

**Figure 9:**
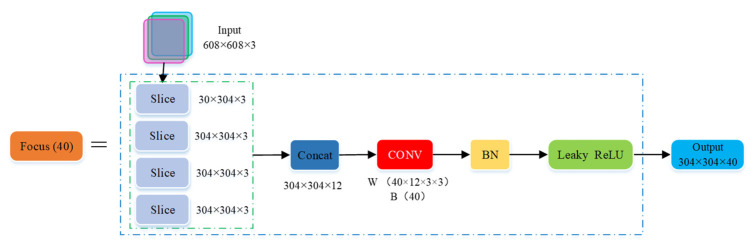
Structure of Focus module.

The first layer of the backbone network is the Focus module (seen in Figure 9), which slices the image and fully extract features to retain more information; its aim is to reduce the amount of model calculations and speed up model training [31]. Its specific structure is as follows: Firstly, the image datasets (three channel picture, the size is 608 × 608 × 3) at the Input terminal were divided into four slices (the slice size was 304 × 304 × 3) using the slice operation. Secondly, the concat operation was used to connect the four slices in depth to generate a feature map (the image size was 304 × 304 × 12). Then, the convolution layer composed of 40 convolution kernels was used for convolution operations to generate a new feature map (the image size was 304 × 304 × 40). Finally, the output results were generated by batch normalization (BN) and leaky ReLU activation function, and output to the CBL module.

**Figure 10:**
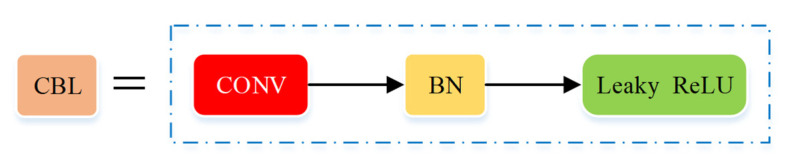
Structure of the CONV-BN-Leaky ReLU (CBL) module.

The second layer of the backbone network is the CBL module (seen in Figure 10), which is the smallest component in the YOLO network structure, the main component of the backbone network and the neck network, and it is mainly composed of the convolution layer, the BN layer and the leaky ReLU activation function, where the number of convolution kernels in the convolution layer determines the size of the output image of the CBL module.

**Figure 11:**
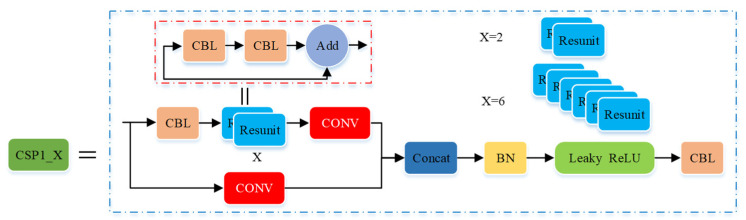
Structure of the CSP1_X module.

The third layer of the backbone network is the CSP1_X module (seen in Figure 11). The CSP1_X and CSP2_X modules are designed drawing on the design idea of CSPNet. The module first divides the feature mapping of the basic layer into two parts, and then combines them through the cross-stage hierarchical structure, reducing the amount of calculation and ensuring accuracy. The CSP1_X module contains CBL blocks and X residual components (Resunit) and aims to better extract the deep features of the image. The value of X represents the number of Resunit, where the residual component is mainly composed of two CBL modules, and its output is the addition of the output of the two CBL modules and the original input. The specific structure of the CSP1_X module is as follows: Firstly, the initial input was input into two branches, and the corresponding convolution operation was performed in the two branches, respectively. Secondly, the output feature maps of the two branches were connected in depth by the concat operation. Then, batch normalization (BN) and leaky ReLU activation function processing were performed. Finally, the convolution operation was performed in the CBL module, and the size of the output feature map was the same as the original input feature map of the CSP1_X module.

**Figure 12:**
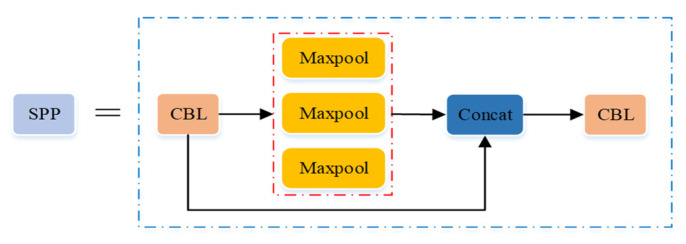
Structure of Spatial Pyramid Pooling (SPP) module.

The ninth layer of the backbone network is the Spatial Pyramid Pooling (SPP) module (seen in Figure 12), which transforms the feather map with arbitrary resolution into a feature vector with the same dimension as the full connection layer, and its aim is to improve the receptive field of the network. Its specific structure is as follows: Firstly, the convolution operation was performed in the CBL module. Secondly, the maximum pooling operation was performed through three parallel maximum pool layers. Then, the feature map after the maximum pooling was deeply connected with the feature map after the convolution. Finally, the convolution operation was performed again in the CBL module.

**Figure 13:**
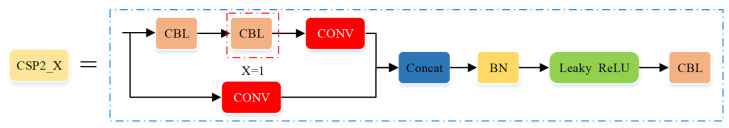
Structure of CSP2_X module.

The first layer of the Neck network is the CSP2_X module (seen in Figure 13). Its specific structure is basically similar to the CSP1_X module, the only difference is that X in the CSP2_X module represents the number of CBL modules. In the improved YOLOv5 architecture, the CSP2_X modules of the Neck network are all CSP2_1.

#### 4.2.2. Improvement of YOLOv5 Network Strategy

In this paper, we mainly improve the YOLOv5 model from two dimensions: one is to use the hidden layer pruning method to adjust the network depth, and the other is to use the convolution kernel pruning method to adjust the network width.

In terms of network depth, we use the hidden layer pruning method to control the number of residual components in the CSP structure to change the network depth; the network depth comparison between the improved YOLOV5 model and the YOLOV5 model are shown in Table 3. It can be seen from Table 3 that compared with other YOLOv5 models, our model is different in the CSP module of Backbone network and Neck network. In the Backbone network, the first CSP1 module has two residual components, the second CSP1 module has two residual components, and the third CSP1 structure has six residual components. In the Neck network, five CSP2 modules have only one residual component. In this way, we can compress the size of the YOLOv5 model, make the model more lightweight under the premise of ensuring the detection accuracy, meanwhile, better extract the depth features of the image.

In terms of network width, we use the convolution kernel pruning method to control the number of convolution kernels in the Focus and CBL structure to change the network width. The network width comparison between the improved YOLOV5 model and the YOLOV5 model is shown in Table 4. It can be seen from Table 4 that compared with other YOLOv5 models, the number of convolution kernels selected by our model in different module structures is different. 40 convolution kernels are used in the Focus module, 80 convolution kernels are used in the first CBL module, 160 convolution kernels are used in the second CBL module, 320 convolution kernels are used in the third CBL module and 640 convolution kernels are used in the fourth CBL module. In this way, we can shorten the width of the YOLOv5 network, improve the object detection speed and the average accuracy.

## 5. Simulation and Experiment Results

### 5.1. Simulation

#### 5.1.1. Training Platform

Based on the HP Pavilion personal computer (Intel (R) Core (TM) I7-9700F CPU, 3.0 GHz, 8 GB memory; NVIDIA Geforce GTX 1080 GPU), the PyTorch deep learning framework was built under the Windows 10 operating system, and the program code written in the Python language is based on the Python3.8 platform.

In this study, the improved YOLOv5 network adopts stochastic gradient descent (SGD) as an optimizer to optimize network parameters. The weight decay was set to 0.0002, the momentum was set to 0.937, the number of iterations epochs was set to 1000, the value of the learning rate was set to 0.001, and the batch_size was set to 64. The data set has a total of 4000 samples, where the training set was set to 3000 and the test set was set to 1000. The batch_size and learning rate are important factors that affect the performance of the model, therefore, we will optimize the parameters of our model to obtain an optimized model with better performance.

#### 5.1.2. Model Simulations 

**Figure 14:**
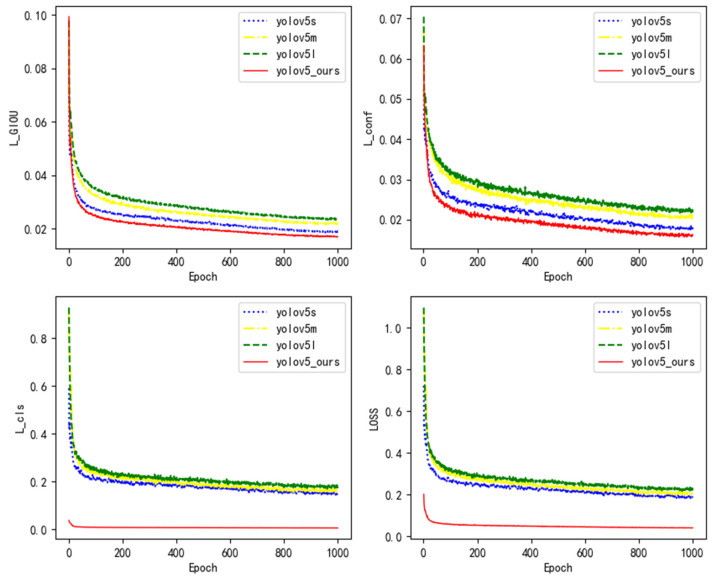
The convergence curve of the loss function for different YOLO models.

YOLOv5x is an extended model of the YOLOv5 series. Its model is relatively large and its calculation speed is slow, which does not meet the lightweight pursuit of grasping robot object detection. Therefore, YOLOv5x model is reasonably abandoned. After the parameters were set, the YOLOv5s, YOLOv5m, YOLOv5l, and YOLOv5_ours were simulated, respectively. The convergence curve of the model loss function is shown in Figure 14.

Based on the simulation results, the following conclusions can be reached.

(1) In Figure 14, the loss function of the YOLOv5l model converges the slowest and the loss value is the largest, followed by the YOLOv5m and YOLOv5s models, and the loss function of the YOLOv5_ours model converges the fastest and the loss value is the smallest.
(2) It can be seen from Figure 14 that the YOLOV5_ours model loss function convergence curve drops the fastest, and the loss value stabilizes after 200 iterations, indicating that the improved YOLOv5 model proposed in this paper has a better object detection effect.

### 5.2. Simulation Analysis

In order to further analyze the recognition performance of the model proposed in this paper, models such as YOLOv5s, YOLOv5m, YOLOv5l and YOLOv5_ours were used to identify the data set. In addition, quantitative analyses of the model with evaluation indicators such as precision, recall and mean average precision were performed. The performance results of different detection models are shown in Table 5.

Based on the simulation results, the following conclusions can be reached.

(1) In Table 5, the precision, recall, mAP value and F1 score of the proposed model were 99.35%, 99.38%, 99.43% and 99.41%, respectively.
(2) It can be seen from Table 5 that the YOLOv5_ours model proposed in this paper has the highest precision and mAP value. The precision value was 1.11%, 1.16% and 1.24% higher than the YOLOv5s, YOLOv5m and the YOLOv5l networks, respectively, and the mAP value was 1.12%, 1.2% and 1.27% higher than YOLOv5s, YOLOv5m and the YOLOv5l network respectively, indicating that the YOLOv5_ours model has the best object detection accuracy among the four methods.
(3) It can be seen from Table 5 that the YOLOv5_ours model proposed in this paper has the highest recall value and F1 score, where the recall value was 1.01%, 1.12% and 1.2% higher than the YOLOv5s, YOLOv5m and YOLOv5l networks, respectively, the F1 score was 1.13%, 1.2% and 1.28% higher than the YOLOv5s, YOLOv5m and YOLOv5l networks respectively, indicating that the YOLOv5_ours model has the best effect on object recognition among the four methods.
(4) It can be seen from Table 5 that the size of the YOLOv5_ours model proposed in this paper is only 12.5 MB. Compared with the original YOLOv5s, YOLOv5m and YOLOv5l models, the scale of the model has been reduced by 10.71%, 70.93% and 86.84%, respectively, indicating that the YOLOv5_ours model can not only guarantee the recognition accuracy, but also realize the lightweight properties of the network effectively.
(5) Overall, the YOLOv5_ours model proposed in this paper has the highest precision, recall, mAP value and F1 score among the four network models; additionally, it has lightweight properties and can be deployed well in embedded systems.

### 5.3. Experiment Results

**Figure 15:**
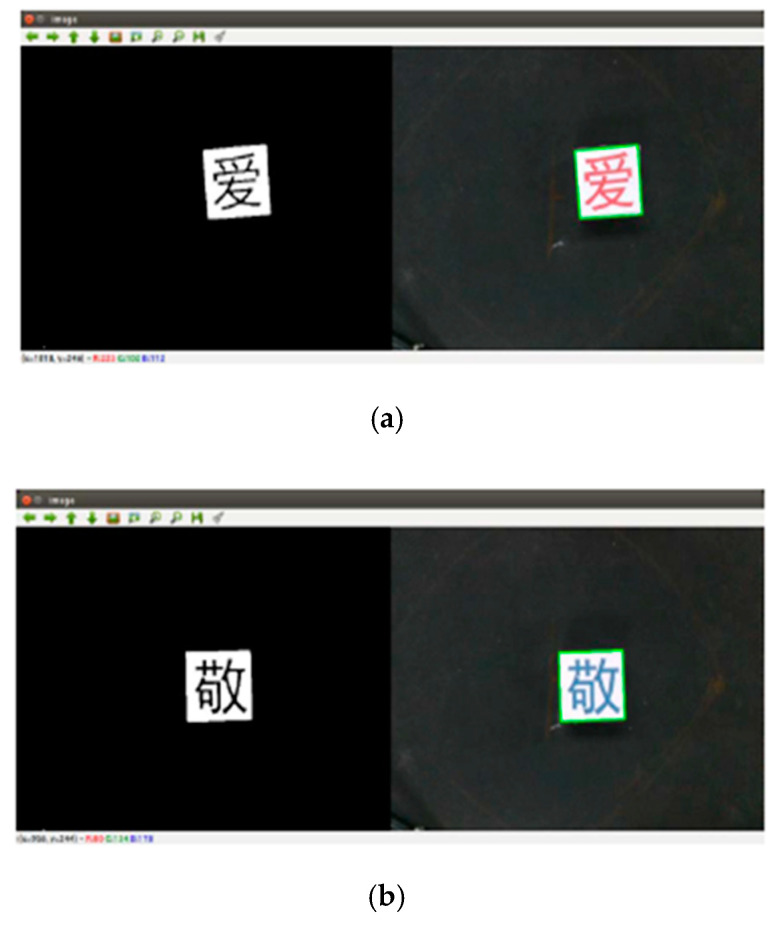

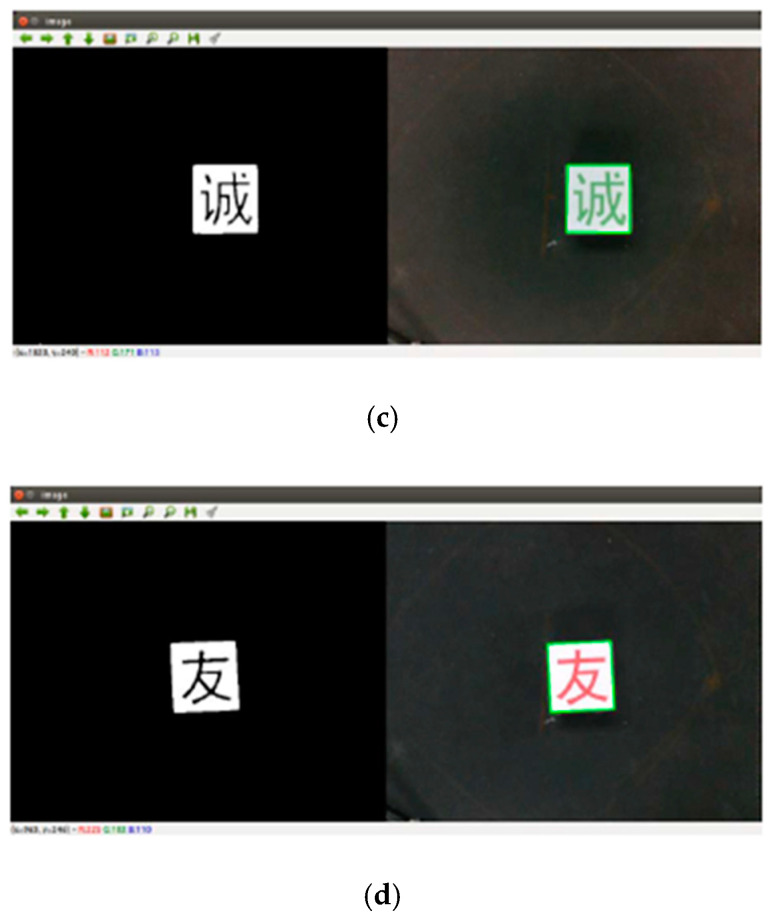
Actual recognition results of object detection. (**a**) Recognition result of Chinese character wooden block “ai”; (**b**) Recognition result of Chinese character wooden block “jing”; (**c**) Recognition result of Chinese character wooden block “cheng”; and (**d**) Recognition result of Chinese character wooden block “you”.

In order to verify the feasibility of the method proposed in this paper, the Xarm robot is used to perform object detection tasks. Firstly, the Xarm robot is used to identify the objects. Secondly, the objects are matched and the objects’ positions are obtained. Then the objects are grabbed from the scattered wooden blocks. Finally, the objects are placed in order. The actual recognition results of object detection are shown in Figure 15.

Based on the simulation results, the following conclusions can be reached.

(1) Figure 15 shows the recognition results of four different Chinese character wooden blocks, where the left side of the image is the actual object detected by the camera, and the right side is the recognition result of the actual object.
(2) It can be seen from Figure 15 that the actual recognition result of the Xarm robot on the wooden block is very clear, and the effective recognition and calibration of the object can be achieved.
(3) It can be seen from Figure 15 that the method proposed in this paper has good object detection accuracy, can be applied to the actual production operations, and has great theoretical research and application value.

## 6. Conclusions

This paper proposes an object detection method based on an improved YOLOv5, which can realize more accurate positioning and recognition of objects by the grasping robot. In the improved YOLOv5 object detection method, the network pruning method is used to optimize the depth and width of the YOLOv5 network, thereby realizing the lightweight improvement of the network, and can be deployed to industrial equipment. Meanwhile, the optimal parameters of the YOLOv5_ours model are determined through optimization experiments on the learning rate and batch_size, and compared with other YOLO series models, the model proposed in this paper has the lowest loss function value.

The results indicate that the precision, recall, mAP value and F1 score of the proposed YOLOv5_ours model were 99.35%, 99.38%, 99.43% and 99.41%, respectively. Contrasting with the original YOLOv5s, YOLOv5m and YOLOv5l models, the mAP of the YOLOv5_ours model has increased by 1.12%, 1.2% and 1.27%, respectively, and the scale of the model has been reduced by 10.71%, 70.93% and 86.84%, respectively.

The research of this paper only collects the wooden block image data set. In the future, other types of data sets need to be made to increase the detection range of the model, so as to be better applied in practical production. In addition, the recognition and grasp of more complex objects under high-speed conditions is also worthy of further study.

## Figures and Tables

**Table 1:** Quantity details of wooden block image datasets.

Data Category	Chinese Character	Letter	Special-Shaped	Punctuation	Blank	Total
Data collection	300	300	300	50	50	1000
Data augmentation	900	900	900	150	150	3000
Total	1200	1200	1200	200	200	4000

**Table 2:** YOLOv5 network structure.

YOLOv5	Features
Input	Mosaic data augmentation, adaptive anchor, adaptive image scaling
Backbone	Focus, CBL, 3×CSP1_X, SPP
Neck	CBL, 5×CSP2_X, Upsample, Concat, FPN+PAN
Head	GIoU_Loss

**Table 3:** Network depth comparison of different YOLOv5 models.

Model	Backbone: CSP1_X			Neck: CSP2_X				
	First	Second	Third	First	Second	Third	Fourth	Fifth
YOLOv5s	CSP1_1	CSP1_3	CSP1_3	CSP2_1	CSP2_1	CSP2_1	CSP2_1	CSP2_1
YOLOv5m	CSP1_2	CSP1_6	CSP1_6	CSP2_2	CSP2_2	CSP2_2	CSP2_2	CSP2_2
YOLOv5l	CSP1_3	CSP1_9	CSP1_9	CSP2_3	CSP2_3	CSP2_3	CSP2_3	CSP2_3
YOLOv5x	CSP1_4	CSP1_12	CSP1_12	CSP2_4	CSP2_4	CSP2_4	CSP2_4	CSP2_4
YOLOv5_ours	CSP1_2	CSP1_6	CSP1_6	CSP2_1	CSP2_1	CSP2_1	CSP2_1	CSP2_1

**Table 4:** Network width comparison of different YOLOv5 models.

Model	Number of Convolution Kernels				
	Focus	First CBL	Second CBL	Third CBL	Fourth CBL
YOLOv5s	32	64	128	256	512
YOLOv5m	48	96	192	384	768
YOLOv5l	64	128	256	512	1024
YOLOv5x	80	160	320	640	1280
YOLOv5_ours	40	80	160	320	640

**Table 5:** The performance results of different YOLOv5 detection models.

Models	Precision (%)	Recall (%)	mAP (%)	F1 (%)	Model Size (MB)	Train Time (h)	Inference Time (ms)
YOLOv5s	98.24	98.37	98.31	98.28	14	18.532	35
YOLOv5m	98.19	98.26	98.23	98.21	43	19.118	56
YOLOv5l	98.11	98.18	98.16	98.13	95	21.834	77
YOLOV5_ours	99.35	99.38	99.43	99.41	12.5	18.184	32

## Data Availability

Not applicable.
